# Network architectures supporting learnability

**DOI:** 10.1098/rstb.2019.0323

**Published:** 2020-02-24

**Authors:** Perry Zurn, Danielle S. Bassett

**Affiliations:** 1 Department of Philosophy, American University, Washington, DC 20016, USA; 2 Department of Bioengineering, School of Engineering and Applied Science, University of Pennsylvania, Philadelphia, PA 19104, USA; 3 Department of Physics and Astronomy, College of Arts and Sciences, University of Pennsylvania, Philadelphia, PA 19104, USA; 4 Department of Electrical and Systems Engineering, School of Engineering and Applied Science, University of Pennsylvania, Philadelphia, PA 19104, USA; 5 Department of Neurology, Perelman School of Medicine, University of Pennsylvania, Philadelphia, PA 19104, USA; 6 Department of Psychiatry, Perelman School of Medicine, University of Pennsylvania, Philadelphia, PA 19104, USA; 7 Santa Fe Institute, Santa Fe, NM 87501, USA

**Keywords:** network neuroscience, knowledge networks, constraints, curiosity, collective dynamics

## Abstract

Human learners acquire complex interconnected networks of relational knowledge. The capacity for such learning naturally depends on two factors: the architecture (or informational structure) of the knowledge network itself and the architecture of the computational unit—the brain—that encodes and processes the information. That is, learning is reliant on integrated network architectures at two levels: the epistemic and the computational, or the conceptual and the neural. Motivated by a wish to understand conventional human knowledge, here, we discuss emerging work assessing network constraints on the learnability of relational knowledge, and theories from statistical physics that instantiate the principles of thermodynamics and information theory to offer an explanatory model for such constraints. We then highlight similarities between those constraints on the learnability of relational networks, at one level, and the physical constraints on the development of interconnected patterns in neural systems, at another level, both leading to hierarchically modular networks. To support our discussion of these similarities, we employ an operational distinction between the modeller (e.g. the human brain), the model (e.g. a single human’s knowledge) and the modelled (e.g. the information present in our experiences). We then turn to a philosophical discussion of whether and how we can extend our observations to a claim regarding explanation and mechanism for knowledge acquisition. What relation between hierarchical networks, at the conceptual and neural levels, best facilitate learning? Are the architectures of optimally learnable networks a topological reflection of the architectures of comparably developed neural networks? Finally, we contribute to a unified approach to hierarchies and levels in biological networks by proposing several epistemological norms for analysing the computational brain and social epistemes, and for developing pedagogical principles conducive to curious thought.

This article is part of the theme issue ‘Unifying the essential concepts of biological networks: biological insights and philosophical foundations’.

## 1. Introduction

The human mind is equipped with rich materials and diverse strategies with which to interpret flows of information into structured units and relations [1,2]. In many cases, such interpretive inferences are drawn from temporally extended streams of stimuli where bits of information are presented to our perceptive apparatus in a sequential manner [3–8]. We read a book or listen to a lecture composed of word sequences. We listen to a song or instrumental piece composed of sound sequences. We engage in discussions with sequential arcs. We perceive a visual scene composed of light and colour sequences. We walk through the day and experience sequences of heat, air currents and human touch. From those one-dimensional streams of information we infer the complex structure of the world, and our potential knowledge thereof [9,10].

In fact, reality is knowable as a set of informational units and relations among them. It is these units and their relations that scientists devote their lives to understanding. Henri Poincare noted in his book *Science and Hypothesis* (1902) that, ‘The aim of science is not things themselves, as the dogmatists in their simplicity imagine, but the relations among things; outside these relations there is no reality knowable.’ [11, p. xxiv] While Poincare’s post-Kantian claim may seem eminently reasonable and straightforward, its implications are perhaps more intriguing than he knew. If science is concerned not with reality itself, but with the relations among things, then scientific knowledge relies on perpetually fine-tuning the network architectures of information. That is, what can be known is a network of relations, and knowledge itself is a network of that information. John Dewey in his book *Democracy and Education* (1916) suggested as much when he wrote that, ‘[K]nowledge is a perception of those connections of an object which determine its applicability in a given situation. [· · ·] An ideally perfect knowledge would represent such a network of interconnections that any past experience would offer a point of advantage from which to get at the problem presented in a new experience.’ [12, p. 116] Scientific knowledge, then, is an increasingly effective network of ideas that model interconnections in the world.

And what is the apparatus by which we process and construct the relations between things and reason through those relations (i.e. engage in relation and relational learning) [13–15]? Our primary computational infrastructure is the brain [16]. Much of what we now know about the brain relates to the putative functions of single areas, and has been derived from lesion and imaging studies in both human and non-human animals [17,18]. Yet, recent work has noted a marked increase in explanatory power from circuit-level descriptions that map the location, transmission, and manipulation of information throughout spatially distributed networks of neural units [19–21]. Over the past decade or more, the study of the network architecture of neural circuits has been formalized in the field of *network neuroscience* [22], which draws on graph theory, statistical mechanics and network science to create and study network models of neural systems [23–25]. In some ways, this appreciation of the brain as a networked system is new, particularly in its formal mathematical nature; yet in other ways, this appreciation is simply a remembrance of what we have speculated about for almost two centuries since Schwann’s proposal in 1839 [26], and known decisively for more than one century as the Neuron Doctrine [27]. In 1906, Cajal and Golgi were awarded the Nobel Prize for Physiology or Medicine for their demonstrative experiments confirming that nerve cells are the discrete units that make up brain tissue, and that they comprise a connected network system by discrete sites of contact.

We pause at this juncture in the ever-vigorous progress of scientific and philosophical investigation into the nature of mind and reality. We focus our attention on the networked nature of knowledge as well as the networked nature of the computational unit—the brain—that allows the human mind to process and construct that knowledge. We review recent evidence for network constraints on learnability of information and network constraints on the architecture of neural circuits that support that learning. We discuss similarities in those constraints and attempt to reason about why there might exist a marked correspondence in hierarchically modular organization in both types of networks. Our discussions of the architecture of knowledge, the architecture of the brain, and their relations allow us to then explicitly reason about the relationship between the modelled, the modeller, and the model. That reasoning leads us to ask how recent empirical evidence could inform deeper explanations and mechanisms for knowledge acquisition. We engage in an interdisciplinary discussion of possible epistemological norms for studying brain network architecture and its role in the acquisition of knowledge networks. Finally, we close with a few thoughts on how these discussions could inform pedagogical principles conducive to curious thought engendering knowledge acquisition. Because we come from quite different areas of inquiry (philosophy, physics and neuroscience), and because we hope that this piece will be accessible across fields, we aim for a simple and clear presentation of the ideas, and eschew jargon wherever possible. We have been free with our citations to ensure that practitioners in a given field are directed to relevant work in their disciplinary domain. Notably, what we provide is a review of extant literatures, selected for their relevance and insight into the network architectures supporting learnability, upon which future experimental and theoretical analyses might be built.

## 2. Network constraints on the learnability of relational knowledge

To make our discussion here a bit more concrete, let us consider a professor sitting down at their (likely disheveled) desk to develop tomorrow’s lecture or discussion plan. For simplicity, let us ignore the precise topic of the lecture or discussion (and the related problem of how students learn representations useful for modelling the world), and instead focus solely on the structure of the content. Perhaps the content can be quite easily subdivided into 15 narrowly defined concepts, which are related to one another in a non-trivial topology. Concepts 1 through 5 may be strongly related, forming a module; concepts 6 through 10 may be strongly related, also forming a module; and concepts 11 through 15 may be strongly related, forming a third module. But the three modules are not completely independent of one another; instead, module 1 is conceptually linked to module 2, and module 2 is conceptually linked to module 3, which in turn harks back to module 1. How does the professor choose to take this potentially high-dimensional network architecture between concepts, and translate it to the students when time is one-dimensional and uni-directional, and thus only one word can be spoken at a time, and presumably only one concept presented or discussed at a time? The same challenge is faced by any writer or speaker: how must one take a bit of knowledge, with some inherent network architecture of relations between informational units, and translate that knowledge into a continuous stream of words? Will the reader or listener or discussant be able to infer the pattern of relations between units? If so, how? Is there an optimal mapping of the network into a stream that supports rapid inference on the part of the receiver or interlocutor?

### (a) Statistical learning and the relevance of transition probabilities

Broadly, the problem of inferring patterns of pairwise dependencies from incoming streams of data is in fact much more general than simply listening to a lecture or engaging in a discussion. Indeed, the capacity to make such inferences allows us to learn language [28], segment visual events [3], parse tonal groupings [4], parse spatial scenes [5,29], infer social networks [6,30] and perceive distinct concepts [7,8,31,32]. The underlying general learning mechanism is known as *statistical learning*, which can be defined as the ability for humans and other animals to extract statistical regularities from the world around them to learn about the environment [33]. For example, a baby can listen to a stream of syllables and detect the probabilities with which syllables follow one another. Sets of syllables that follow one another with high probability are perceived as units (such as words); when one syllable rarely follows a second syllable, the transition is perceived as a boundary between units (a break between words). Although first identified in human infant language acquisition, statistical learning is now thought to be a generalized learning mechanism that is relevant across information modalities and operationalized in multiple species [34].

### (b) Moving beyond local transition probabilities

While it was clear from its inception that statistical learning offered a compelling description for sensitivity to pairwise dependencies between informational units, it was not immediately clear whether that description could be extended to explain sensitivity to a complex network structure underlying sequential input from our world [35]. The foundational work in statistical learning manipulated the transition probability between two adjacent stimuli in a temporal stream. Yet, evidence quickly accumulated that supported the notion that humans were also capable of learning from the probabilities between non-adjacent stimuli [36,37], quaintly referred to as ‘learning at a distance’ harking back to the quantum mechanical notion of ‘action at a distance’ [38]. For example, we come to know not only that ‘Peter’ and ‘Rabbit’ are distinct words, but also that we are more likely to see or hear those two words in the same story than to hear ‘Thayne’ and ‘Rabbit’ in the same story. Human sensitivity to structure beyond adjacent transition probabilities was further underscored by pioneering work from Schapiro and colleagues, who drew a sequence of visual stimuli from a random walk on a network while keeping all transition probabilities fixed at a constant value [39]. The network contained three main modules and the investigators observed that humans were able to demarcate module boundaries from the temporal stream, supported by neural activity in the hippocampus [40].

### (c) Explicitly probing learnability of network architectures

Following these important studies that provided initial suggestions that humans were sensitive to a network architecture guiding the statistics of their experiences, the field faced two main challenges. First, an experimental paradigm was needed that could provide an assessment of exactly how much each relation (or edge) in the network was learned. Like Shapiro *et al.* [39], Karuza and colleagues used a task in which a stream of visual stimuli was constructed by traversing a given network using a particular type of walk and in which humans were given a cover task of detecting whether stimuli were upright or rotated [41]. From the cover task, the investigators were able to extract a reaction time for each transition between two stimuli; from the type of walk (random, Eulerian and Hamiltonian), the investigators were able to determine that the manner in which the network was traversed impacted human expectations. Second, the field needed a clear demonstration that human expectations could be manipulated differently by different network architectures. Kahn and colleagues studied human expectations derived from a stream of stimuli drawn from a random walk on three different network architectures (modular, lattice and random), and showed that humans reacted with differential swiftness to sequences constructed from each network type [42]. The work also mapped the original context of visual stimuli [6,39,41] to motor commands, thereby demonstrating that network learning was robust across modalities.

### (d) Hallmarks of network learning in humans

Throughout the existing literature, the human capacity to acquire expectations about a network architecture underlying a temporal stream of information is particularly marked by the so-called cross-cluster surprisal [41]: humans react more slowly to transitions between modules in a network than to transitions within modules [6,30,41–43]. This finding suggests that humans are able to infer the presence of higher dimensional topological clusters within one-dimensional streams of information. As a human behaviour, this effect on reaction time is particularly striking in light of the fact that the transition probabilities of all edges are identical, indicating that humans must be sensitive to a meso-scale or global organization, unfolding over long time scales within the information stream. Perhaps even more strikingly, humans react more swiftly when the stream of information is drawn from a modular network than when it is drawn from a lattice network [42,43]. In turn, this behaviour suggests that humans find the modular architecture relatively easy to learn, although it is as yet unclear whether that ease is explained by innate knowledge of certain graphical motifs [1], a flexible learning algorithm [44–46], or constraints on the computational complexity of associated cognitive processes [43,47]. The human response to network-based temporal streams of information is remarkable when we consider the mental computations that subserve it. Neither the cross-cluster surprisal effect nor the modular-lattice effect would be observed from simulated agents with optimal rationality, who instead would accurately learn the transition probabilities that are held constant across all edges and all network architectures in these experiments.

### (e) Building mental models of our world

To explain these curious, non-artificial (some would even say non-optimal) features of human behaviour, we turn to the question of exactly how humans build models of their world. While this question has been asked in different ways for millenia [48], and from within the discipline of psychology for decades [1,2], here we focus on the specific question of how humans perceive relational knowledge, building models of network architectures explaining transition probabilities of sequentially experienced stimuli. We consider the relatively reasonable hypothesis that humans seek to minimize both computational resources and errors, which can be formalized by the free-energy principle [43]. We then draw on a subfield of theoretical physics known as statistical mechanics to stipulate a maximum entropy (minimal complexity) solution, thereby blending principles of thermodynamics and information theory. The formal mathematical model explains human behaviour by predicting that humans perform a sort of fuzzy temporal integration, which serves to strengthen their expectations of edges in local clusters. Using this model, we can account for both the cross-cluster surprisal effect and the modular-lattice effect in current human experiments, and we can further predict human responses to arbitrary network architectures [43]. In exercising the model on simulated data, we expect that humans will be able to learn information most swiftly and accurately on hierarchically modular networks [49], a prediction that can be directly tested in future experiments in both real and artificial learning systems [50]. But first, we turn to the biological apparatus that allows network learning to occur.

## 3. Network constraints on interconnection patterns in neural systems

As our professor sits down to their dishevelled desk to prepare tomorrow’s lecture or discussion plan, they may or may not consider the learning organ in the minds of their students. That organ—the human brain—is a richly structured apparatus that has been built to model the world. The acts of building have occurred slowly over evolutionary time scales, and are also modulated within an organism’s lifetime by developmental programmes as well as the prevailing forces of the local environment. As with any remarkably useful tool, there exists a systematic map between the physical architecture of the brain and the functions made possible by that architecture. This is not to say that a single function can only be supported optimally by a single structure [51,52], but instead to say that there exist constraints on the class of structures that can or cannot support a given function [53,54]. For example, the structure of synapses between neurons in the nematode *Caenorhabditis elegans* allows for motoric capabilities and mechanized action [55], while the structure of primary afferent connections in the *Drosophila* olfactory system explains odour lateralization behaviour [56]. Similarly, in the human, the connection pattern of white matter tracts linking large-scale brain areas allows for information flow between visual and motor cortices supporting motor skill acquisition [57]. While collating these discrete observations can be useful, it would arguably be more satisfying to identify broad principles that can serve to parameterize the relation between structure and function in neural systems. Here, we briefly review the literature on the network structure of neural systems, and the clear constraints upon it.

### (a) Energy expenditure and metabolism

The brain evolves, develops and functions under constraints on energy expenditure [58–60]. Early work noted that the shape of neuronal arbours appears to be explained by a minimization of wiring, which in turn minimizes the energy required for synaptic communication in local neural circuits [61,62]. At resolutions larger than the subcellular scale, the principle of wiring minimization also explains why the layout of ganglia in the nematode nervous system requires the least total connection length out of 40 000 000 alternative layouts [63]. Wiring minimization may be balanced by constraints that are topological in nature; for example, early evidence in the rhesus macaque demonstrated that neural networks are more similar to network layouts that minimize the length of processing paths, rather than the length of wires [64,65]. In a sparse network, processing paths allow two units that are not directly connected to nevertheless communicate across a string of direct paths between serially ordered intermediate units. Minimization of physical lengths or of processing paths leads to a network topology marked by (i) strong local clustering, supporting local processing and (ii) short average path lengths from any point in the network to any other point, supporting global processing [66]. The combination of local clustering and short path lengths is consistent with existing models of small-worldness [67], which in turn are associated with efficient communication in many informational systems spanning technology [68], physics [69], linguistics [70] and biology [23,71].

### (b) Information processing and computation

It seems sensible to state that optimal information processing requires both local and global components, but it is unclear whether those two constraints are sufficient to produce ideal neural systems [72,73]. Let us consider information transmission as distinct from processing, and note that reasonable architectures to support transmission are bipartite structures [74–76], in which a set of network nodes are strongly and preferentially connected to another set of network nodes, but nodes within a set are not connected to each other [77]. Such bipartite connectivity is observed in neural networks across *C. elegans*, *Drosophila*, the rhesus macaque, the mouse and the human [78], and offers utility in predicting how the activity of neural systems responds to perturbations [79]. Next, consider the potential necessity for information broadcast and receipt; these processes are best supported by core-periphery architectures [80], in which a densely intra-connected set of nodes (the core) extends connections to a sparsely intra-connected set of nodes (the periphery). Core-periphery organization is noted in the structural networks of neural systems across several species [78] as well as in functional brain networks in humans [81–84], allowing for broadcast and receipt functions [85], error prediction [86] and adaptation during learning [87]. Together, small-world organization, bipartitivity and core-periphery structure allow for a diverse array of informational processes that could support the function of neural systems as modellers of our world.

### (c) Evolution, development, adaptation and learning

A key feature of neural systems that is *not* directly explained by the constraints and structural motifs described thus far is their capacity to evolve, develop and adapt. Evolutionary theory suggests that such adaptibility is made possible by structural modularity [88,89], which arises naturally in systems that must satisfy different goals in a changing landscape [90,91]. Moreover, work in both evolutionary biology and evolutionary computer science [92] suggests that hierarchical modularity—the recursive composition of submodules—arises naturally in these same systems when they evolve under constraints for wiring minimization [93]. Hierarchical modularity has been described as the generic architecture of complexity [54], and is observed beyond the neurosciences, in metabolic, ecological and gene regulatory networks, and in human-made systems, such as large organizations and the Internet [93]. The current structure of the human brain is a reflection of evolutionary pressures to optimize neural function and constraints from what other systems and capacities had already developed at each stage of evolution; recent studies suggest that these pressures and constraints naturally guide the system towards hierarchical modularity [93–98]. In the human brain, hierarchical modularity has been noted in the structural networks linking large-scale areas [65] and in functional networks linking these same areas by shared information [99].

From a psychological perspective, hierarchical modularity is a natural substrate for the separation of cognitive processes [100] and a conduit for the specialization of function in distinct volumes of neural tissue [101]. Yet, it is important to note that not all of the specifics of the early ideas of cognitive or mental modularity withstood the test of time or deeper scientific investigation [100–103]. Those early ideas have been altered and fine-tuned in the light of new empirical data and the capacity to test such theories across large cohorts, for example, in the more than 1000 humans who participated in the Human Connectome Project [104,105]. A recent study used an author-topic model of cognitive functions across 9208 experiments of 77 cognitive tasks to demonstrate a strong spatial correspondence between cognitive functions and brain network modules, suggesting that each module performs a discrete cognitive function [106]. A subsequent study further suggested that specific brain regions tune the connectivity of their neighbouring regions to be more modular while allowing for the integration of task appropriate information across communities, in a manner that facilitates cognitive performance [107]. Such studies lend support to the notion that a map between cognitive modularity and brain modularity does in fact exist, but its specific form may be different from that postulated several decades ago. The existence of such a map also suggests that adaptible brain modules may support adaptible cognition. Indeed, the predicted support of modularity for adaptibility is particularly evident in recent work demonstrating that the modules within functional brain networks flexibly reconfigured over time in support of human learning [108–111], planning and reasoning [112], and cognitive flexibility [113]. From a theoretical perspective, the relation between network modularity and adaptible function can be understood in a more mechanistic manner by considering the fact that network architecture directly constrains the trajectories that a system can take through the adaptation landscape [114].

## 4. Similarities in constraints, leading to hierarchically modular networks

If the professor we have been following understood the architecture of the brain, would that understanding change how they chose the content and structure of their lecture or discussion plan? Most experts could describe, if asked, the direct relations between any pair of the 15 concepts they chose to cover in the class period. In other words, the expert could see the topic as a fully connected graph if they wished; they have all of the requisite knowledge. Yet, an expert can also crystallize that fully connected graph into a sparse network or spanning tree when they wish to use it, or to communicate it; a fully connected network is unlikely to be particularly useful or particularly easy to communicate or apprehend. Which set of important links between ideas should be chosen? Which are sufficient to find a path that connects any pair of ideas in the domain? Should the network architecture of knowledge to be transmitted and the network architecture of the brain inform one another, and if so how and why?

The question brings to mind a passage from Aristotle’s *Metaphysics*, where he considers precisely what happens to the mind when it contemplates. He writes, ‘Mind thinks itself because it shares the nature of the object of thought; for it becomes an object of thought in coming into contact with and thinking its objects, so that mind and object of thought are the same.’ [115, p. 1072] While the notion that mind and object of thought are the same might initially appear fanciful and rather arcane, there are many metaphors and research programmes that reflect the human intuition that there exists some structural similarity in how we think about knowledge architecture and brain architecture. Moreover, emerging evidence offers preliminary support for one candidate operationalization of precisely the notion that mind and object of thought are intimately connected [39,40,116–118]. When a mind is shown relational knowledge with a specified network architecture, brain activity reflects that architecture in a particular manner. Specifically, the pattern of activity in response to a given item (network node) is similar to the pattern of activity in response to another item (network node) to a degree dictated by the topological distance between the items in the network [117,118]. One could think of this form of representation as one in which the brain represents the inter-item distance as a particular type of relation encoded as a node itself in a labelled graph. In fact, humans appear to organize conceptual knowledge in the brain in a manner that is similar to how they organize spatial knowledge [116], coding topological paths akin to physical distances [40,117]. Suppose that this process of producing patterns of activity whose relations match the relations of the items they represent (or the parts of the world they model) occurs consistently over a human’s lifetime, and in fact also over the course of evolution; then what architecture might most effectively underlie the active units to optimize this process?

### (a) Concordance between modeller and modelled

The terms reflect, model, process, represent and encode are distinct and can each separately help us to understand whether and when the modeller and the modelled are somehow concordant. Does a good apparatus display a form that reflects the form of the material on which it works? Not always; the apparatus for Millikan and Fletcher’s 1909 oil-drop experiment has a form far from that of the electron that it is meant to measure [119]. Does a good modeller display a form that reflects the form of the subject to be modelled? Also not always; a 3D printer has a form unlike all of the models that it can build, barring one (itself). Does a representer display a form that reflects the form of the represented? Sometimes; the form of a stationary artist in Times Square or an actor in the West End is the same as the form they represent (although for an alternative view see [120]). Does a processor display a form that reflects the form of the processed? Perhaps; very large scale integrated circuits in computer chips display hierarchically modular structure [65,121], consistent with the structure of information that the chips will represent, manipulate, and store [122]. Does an encoder display a form that reflects the form of the encoded? Efforts in the field of artificial neural networks are continuing to develop architectures and models that can encode the features (both categorical and non-categorical) of an image across hierarchical layers of the neural network. In some cases, the encoding in the artificial system maps to the structure in the real image in an interpretable way [123]: for example, a high-resolution feature is encoded in early layers and a low-resolution feature is encoded in later layers [124–126].

Broadly, across modelling, representing, processing and encoding, the relation between the *er and the *ed can differ. Thus, whether it is happenstance or meaningful that brains, learnable networks and knowledge structures have modular architecture depends upon the nature of the relation between brain and knowledge. Much of the current thought in neuroscience and psychology builds upon the notion that the brain’s principal purpose is to model [127–134]. Thus, a discussion of the architecture of the brain and the architecture of knowledge would be impoverished without a discussion of the relation (the act of modelling) that can formally link the two. And notably, that relation remains to be clarified; decades of prior work demonstrate that it is non-trivial to successfully represent relational structure in neural systems [14,135–143]. Such representations may depend upon the nature of the relations or the content being related, and may manifest distinctly in the scale accessible to fMRI compared to the scale accessible to cellular imaging. Finally, such representations may also differ across regions [118], being precise reflections of the graph or more akin to a predicate logic.

### (b) Correspondence by relation versus by shared constraint

Does correspondence in architecture tell us something important about the nature of modelling in the brain, thus offering hints regarding explanations and mechanisms for knowledge acquisition? There may exist multiple reasonable answers to this question, and those answers might depend on the specific brain area(s) whose architecture we are considering. Is the given brain area (or the entire brain) a modeller, representer, processor, encoder, or all of the above? First, note that to the degree that the brain *represents* knowledge, correspondence between the network structure of neural representations and the network structure of object relations is perhaps expected based on recent empirical studies [39,40,116–118] (although note that further studies are needed that explicitly compare the neural representations developed in response to different network structures). Second, to the degree that the brain *processes* information, correspondence between the network structure of informational connections and the network structure of the information is also perhaps expected [65,121]. These two correspondences come about owing to the nature of the functions *represent* (‘depict’, ‘constitute’, or ‘amount to’ [144]) and *process* (perform a series of mechanical or chemical operations on something in order to change or preserve it [144]). In both cases, a function can lead to a correspondence in the architecture of the modelled and the modeller. But is the reverse inference accurate? If a correspondence exists in architecture between the modelled and the modeller, can we conclude that the correspondence is owing to a functional relation? Not necessarily. Perhaps the simplest counter example is that a modeller can come into existence under similar constraints to the modelled; in this case, the correspondence in architecture is owing to shared constraints rather than a functional relation.

### (c) Concordant versus discordant constraints

Do there exist shared and divergent constraints on network architecture in the brain and in knowledge (or in the reality to which knowledge maps)? Both the world around us and the world within us must obey the laws of physics, and therefore exist under marked constraints on energy and tendencies towards entropy. The pressures specifically for wiring minimization—both to conserve energy and to remain adaptable in a changing environment—are pervasive across both natural and human-made systems from genetic regulatory networks to the Internet [93]. Both the brain and the world around it must maintain robustness over evolutionary time scales, a constraint that could explain their shared modular structure [145], and the redundancy evident in distinct elements serving similar functions within the network [146]. Yet the world and the human brain may not be constrained by all of the same factors; while the human species (and therefore the human mind) must reproduce, must the world reproduce? Or must knowledge reproduce? Moreover, knowledge of the world is not exactly the same as the world itself, and therefore the constraints that impinge on the nature of the world might not always perfectly map onto the constraints that impinge on the nature of knowledge. Any discordances in constraints between knowledge networks and brain networks could explain differences in their architecture or function. But perhaps more importantly, divergence between cognitive constructs and neural instantiations could also allow the two systems to function independently; perfect isomorphisms in the topology of two interconnected networks induce system fragility and vulnerability to control.

## 5. Epistemological norms for analysing neural and social epistemes

Are the constraints impinging upon a brain network or a knowledge network relevant beyond a single individual? Certainly, there exists a distinction between individual knowledge and collective knowledge, and a distinction between brain networks and social networks. While we grant the distinction between these entities and the often different analytics required to study them, their interdigitation is crucial to the advancement of relevant scientific and philosophical inquiry. Here, we extend the discussion of individual knowledge patterns and practices to relational and collective knowledge, in keeping with the contemporary philosophical turn towards social epistemology [147,148] and network epistemology [149–151]. These fields take, as their point of departure, the recognition that an individual knower cannot ultimately be isolated from the social environments in which that knower is said to know. Moreover, we extend the discussion of brain networks to social networks, in keeping with the contemporary neuroscientific turn towards social neuroscience [152] and population neuroscience [153]. These fields recognize that individual brain networks shape social networks [154], that social ties in turn shape the brain [154], and that collective knowledge can alter individual cognition, from attentional capacities and memory processes to social perceptions and decisions [155]. We begin our interscale discussion with epistemology.

### (a) An expanding epistemology

Traditional epistemology, as crystallized by reigning accounts in twentieth-century analytic philosophy, makes some assumptions that, while useful under certain conditions, are no longer considered adequate to our epistemic realities. These assumptions include that knowledge is (1) the purview of an individual human (2) whose beliefs, intentions and propositional attitudes are a critical component of that knowledge. Today, however, it is increasingly important—not to mention useful—to recognize not only the presence of non-human and/or machinic knowers [156,157] and the reality of group or collective knowing [158,159], but what might be called *extended knowing* [160] that traverses knowers of different species, system dynamics and social structures. Such a recognition necessitates, on the one hand, redefining knowledge not as an individual human’s justified true belief [161] but in a more generalizable sense as an evidentially supported explanatory model of some elements of a system [149]. Given the ways in which these models are shared, as well as co-constructed, it is equally important to grapple with the biases implicit in knowledge models in both organic [162] and computational systems [163,164]. These various tasks of revisioning epistemology are largely undertaken by the recent subfields of network epistemology and social epistemology. Building on social epistemology’s insights into the constitutive effects of social relations, investments and institutions on knowledge itself [147,165–167], network epistemology uses formal network theory to elucidate those constitutive effects [148–150,168–170]. Together, network and social epistemology provide a systems-level approach to the processes of knowledge production, as well as the structural limitations of those processes.

### (b) From representation to network architecture

It is largely recognized, across epistemological literature and the history of science, that knowledge neither resembles nor represents, in the technical sense of these terms, things as they are, but rather interprets and constructs things as they are experienced [171–175]. Network epistemology reframes knowledge as a practice of system modelling or network building. As such, it applies a new frame to classic epistemological issues, including the nature of content and testimony [149], consensus [168], communication structures [150], factionalization [151], belief diffusion [148] and curiosity [9,10]. Understanding knowledge as an increasingly effective network of ideas that models interconnections in the world does not preclude standards of efficacy in knowledge network construction or of elegance in the knowledge network architecture, nor does it preclude standards of correctness in knowledge network acquisition or of effectiveness in knowledge network communication. Network epistemology simply extends the epistemic systems under consideration and the questions that can be asked of them. When defining epistemological norms within this framework, for example, it is important not only to attend to structural characterizations, but also functional and causal characterizations. That is, we must ask, ‘What is the architecture of the network?’ but also ‘What is the function of the network’ and ‘What are its causes?’ Such causes are not always perfectly explained by the system’s function, but they can instead be explained by other forces from the system’s environment. In computational, collaborative systems, such as brains, computers and human or non-human collectives, questions of function can be explainable as much by optimization requirements as by suboptimal protocols [150,151].

### (c) Model–modeller–modelled

Let us consider an operational distinction between modeller, model and modelled in the context of our topic of interest. Consider the ‘modeller’ to be that which models (e.g. the brain), the ‘model’ to be that which the modeller makes (e.g. the representation of knowledge that the brain produces) and the ‘modelled’ to be that which is modelled by the modeller (e.g. the information or knowledge present in the human experience). If the brain is taken to model the world, it is incumbent to identify the model–modeller–modelled relationship by which it does so. On the one hand, the form of the model, modeller and modelled may be the same; an example is the actor in the West End. The modelled is Hamlet, the modeller is an actor, and the model is the acted Hamlet. This type of modelling brings to mind the following passage from Rosenblueth & Wiener in their 1945 article in the journal *Philosophy of Science*: ‘That is, in a specific example, the best material model for a cat is another, or preferably the same, cat.’ [176, p. 320] On the other hand, the form of the model and modelled may be the same, but the form of the modeller may be different; an example is the 3D printer. The modelled is a tree and the model is a tree, but the printer is in no way a tree. What is the best way to categorize the brain, as it builds models of the world by learning knowledge networks?

Any discordant constraints between the two systems might lead us to posit that the model–modeller–modelled relationship that we are facing is of the second sort, where modeller is different from model and modelled. But let us consider for a moment whether we see any evidence for the first sort, where model, modeller and modelled are in some meaningful sense the same. Consider that an optimal learning system (the brain) has a modular architecture that allows it to adapt and change, which is the fundamental essence of learning. And what is the system learning? For a moment, let knowledge refer to the knowledge network present in a single mind; it is a subgraph of the Knowledge network extended across that individual’s society, which is in turn a subgraph of the **K**nowledge network present in the combined humanity of today and yesteryear. Collective knowledge can be viewed as a complex system that also must be able to adapt and change; when we find a new piece of information, it must be possible to add it to the knowledge network without rebuilding the system from scratch. Otherwise, knowledge would not serve its purpose, which is to illuminate the ‘veil interposed between reality and the eye of the [mind]’ [177], allowing humanity to interact with the world while not perceiving it fully. To the degree that collective knowledge is an adaptable complex system, it must display modular architecture for precisely the same reason that the brain displays modular architecture. Thus, we have evidence for the first type of model–modeller–modelled relationship.

### (d) Systemic, network and modular bias

From the vantage point of social network epistemology, what is known and reflected in a single brain or a network of brains, in a single computational device or a network of computational devices, will never be simply the result of immediate interaction with perceptions, bits, or data points, but always also with structural limitations and sedimented frames [171,178,179]. Knowledge that is created, shared and distributed across network systems will always reflect the history, goals and limitations of those systems. This modular bias is multi-dimensional and multi-vector. In additionally manifesting evolutionary demands across time, modular bias will manifest current and local demands on organic and inorganic systems, as well as competing goals and epistemic factions. It will also evidence perspectival limitations, including but not limited to inherited and/or algorithmic bias [180], stereotypes [181], structured ignorance and other forms of epistemic injustice [182]. To understand and address issues of modular bias in knowledge network systems and synaptic communication requires increasingly robust work in the politics of human and artificial intelligence, particularly focused on social equity and educational justice.

## 6. Pedagogical principles conducive to curious thought

Developing a deeper understanding of the network architectures of knowledge and knowledge-processing systems such as the brain is of interest in its own right. More than a satisfying intellectual exercise, however, the acquisition of such understanding has the potential to inform and transform our learning environments. As an extension of the robust educational literature exploring the relationship between knowledge networks and social networks [183–187], we posit the relationship between knowledge networks and neural networks as a new pathway for individualizing, optimizing and diversifying pedagogical techniques. Equipped with this knowledge, for example, would our professor sitting at their disheveled desk prepare a different sort of lecture, discussion, or neither? How might we use the existing laboratory experiments in network learning [6,30,39–43,49] to guide best practices in how to present or process information in a way that empowers student learning? As a start, we predict that a modular network architecture underlying information transmission will result in better learning than random or lattice-like architecture, based on the swifter human reaction times observed in visual perceptional learning and visual-motor learning tasks. This prediction could be tested in classroom experiments where a lecture is organized around a set of modularly related concepts versus around a set of linearly related concepts. But beyond the networks studied thus far (only 3 out of the possible 805 491 *k*-4 regular architectures of the 10^14^ 15-node 30-edge graphs), it is important to distill the optimally learnable graph [49] and to ask whether it has a topology that is common in language or in nature. Is the architecture of the optimally learnable graph also the architecture of a well-written paper or a well-written textbook that effectively communicates networked knowledge to the reader [188]?

### (a) Individualization of knowledge presentation

In exploring the network architectures supporting learnability, we would be remiss if we did not ask, ‘Supporting learnability for whom, how, and in what contexts?’ In the search to calibrate learnability to different systems, with their unique learning capabilities and developmental trajectories, new queries are incumbent. For example, do different humans prefer to learn information on different graph architectures [43] specifically because of their cognitive apparatus, which in turn is constrained by their underlying neural substrate [57,189]? If so, would presenting information to humans in their preferred architecture enhance learning? In experimental neuroscience, it is increasingly clear that marked individual differences exist in many types of learning and associated cognitive processes, such as fear learning [190], social learning [191], sensorimotor learning [192], language acquisition and processing [193], media multitasking [194] and executive functions [195]. Moreover, it is clear that humans differ in their general statistical learning capacities [196], as well as in their specific network learning capacities [43]. Such different learning capacities, strategies and preferences motivate a careful study of the network architectures of knowledge that are most easily acquired by a given person. Beyond neuro-typical humans, it is possible that those with disabilities, disorders, or other neuro-atypicalities could further benefit from individualization of knowledge presentation. Such a benefit is underscored by the fact that statistical learning as a general mechanism serves as a window into developmental disabilities such as autism spectrum disorder, specific language impairments, Williame’s syndrome and developmental dyslexia [197]. Even more broadly, it is notable that differences and dysfunctions of basic learning mechanisms accompany a wide range of mental disorders including substance abuse, depression and schizophrenia [198]. Future work could seek to explain neuro-typical and neuro-atypical individual differences in network learning by assessing the trajectories of adaptation that are possible from the underlying neural network architecture [114].

### (b) Exemplifying information-seeking

While we frequently learn from information that is presented to us by an external agent whose goal is for us to acquire knowledge, we often learn best when this process stimulates or supervenes upon an internally driven search for information [199–201]. But is this search innate, something we know how to do without any training [202]? Or is it itself learned as we watch our caretakers, our friends and our mentors exemplify curious search [200]? As a set of investigative practices, curiosity is ultimately a tool. Just as animal and human primates deploy hammers as physical tools [203,204], so they use curious search as an intellectual tool. In the context of pedagogy, we need to investigate how curious search can be both facilitated in students and exemplified by instructors. Whether through lectures, group discussions, hands-on activities, or student research, curiosity can be motivated and modelled [9]. From a network learning perspective, instructors can facilitate student curiosity via a random walk search on the knowledge network (moving from disconnected idea to disconnected idea), or a local walk search on the knowledge network (moving from an idea to a tightly related idea). Instructors can also exemplify the richness of other walk topologies (reflecting other curious typologies), such as a Levy walk in which the probability distribution of step-lengths is heavy-tailed [205]. On a flat landscape, the Levy walk can create a small-world network architecture [205]; by contrast, on the existing knowledge network with a non-lattice topology, a Levy walk can create other more nuanced structures [206,207]. By testing the efficacy of different techniques for facilitating and exemplifying patterns of curious thought, we can begin to build a pedagogy that more robustly encourages curiosity, thereby increasing learnability and well-being [208].

### (c) Curious practice as knowledge network building

What is the logical consequence of idiosyncratic information seeking on knowledge networks unfolding over the time scales of months and years? Preferences for seeking information along certain types of relations, or across specific semantic or conceptual distances, will naturally lead to idiosyncratic architectures of knowledge networks in individual human minds [9]. For example, humans who prefer to close triangles (if A is related to B, and B is related to C, then they want to understand how A is related to C) will naturally build a mesh-like knowledge network architecture. It is interesting to ask whether such individual preferences for styles of knowledge acquisition are evident today or across recent millenia. A recent historical study of the Greek, Latin, German, French and English words for *curiosity* from Plutarch to today demonstrated the existence of at least three key types of curious practice, each characterized by a distinct kinesthetic signature [209]. The *busybody* seeks disconnected bits of information similar to trivia, the *hunter* seeks a specific bit of information in a focused, linear search, and the *dancer* seeks information in local neighbourhoods of knowledge space intermixed with leaps (of analogical or other reasoning) to distant knowledge spaces. Each kinesthetic signature produces a distinct network architecture: respectively a network with many disconnected components, a network with chain-like architecture, and a network with local clustering and long-distance connections, leading to small-world modular architectures [9,10]. Evidence from young children learning the English language supports the notion that such learning is most consistent with the last phenotype, being pocked with gaps in knowledge that are later filled [210]. It would be interesting in future work to determine whether different styles of gappy learning relate to different styles of curiosity [211].

## 7. Conclusion

In this review, we considered the network architectures in both knowledge and brain that support learning. We began by reviewing the network architecture of knowledge and discussed empirical evidence from behavioural experiments in humans that different sorts of network architectures are more or less learnable. Then we reviewed the network architecture of the brain, which supports that learning. We discussed similarities and differences in constraints on network architectures in these two systems. As is clear from the fact that the exposition is peppered with questions, much work is still needed in empirical science and in philosophy separately. But perhaps the most exciting prospects lie in interdigitating these two perspectives to guide the field towards a united understanding of the individual and collective mind and its relation to individual and collective knowledge.

## Data accessibility

This article has no additional data.
